# Age-, Sex-, and Puberty-Associated Reference Intervals for Lipid Profile in Iranian Children and Adolescents

**DOI:** 10.1155/2023/9143234

**Published:** 2023-02-24

**Authors:** Nima Montazeri-Najafabady, Mohammad Hossein Dabbaghmanesh, Naeimehossadat Asmarian, Homeyra Rais Pour

**Affiliations:** ^1^Endocrinology and Metabolism Research Center, Shiraz University of Medical Sciences, Shiraz, Iran; ^2^Anesthesiology and Critical Care Research Center, Shiraz University of Medical Sciences, Shiraz, Iran; ^3^Department of Biostatistics, School of Public Health, Isfahan University of Medical Sciences, Isfahan, Iran

## Abstract

Childhood dyslipidemia is considered a major worldwide health issue. Identification of children with dyslipidemia is notably essential for healthcare providers in establishing and releasing recommendations for the management and prevention of future CVD. In the present study, we provided reference values for the lipid profile from Kawar (a city in the south of Iran) cohort of healthy children and adolescents aged 9–18 years. 472 subjects (234 girls and 238 boys) contributed to the current prospective cohort study using a systematic random sample stratified by age. Fasting lipid levels were measured by enzymatic reagents. Dual-energyX-ray absorptiometry (DEXA) was used to evaluate puberty based on the Tanner stages. LMS Chart Maker and Excel software were used to construct the gender-specific reference plots showing the 3, 10, 25, 50, 75, 90, and 97th percentiles of BMI, cholesterol, TG, HDL, TC, LDL, and non-HDL. The outcomes revealed that concentrations of TC, LDL, and non-HDL were drastically greater in girls as compared to boys. TG increased with age in both genders, while HDL, TC, LDL, and non-HDL declined. We also observed that puberty was associated with higher lipid values in boys and girls except for TG in boys. Our study prepared age- and sex-specific reference intervals for the lipid profile in Iranian children and adolescents. Converted to age and gender percentiles, these reference intervals are expected to serve as an effective and consistent tool for doctors to identify dyslipidemia among children and adolescents.

## 1. Introduction

Childhood dyslipidemia is considered a major worldwide health issue. This condition is positively correlated with a higher risk for cardiovascular disease (CVD), metabolic syndrome [1], and obesity [2], and when tracked into adulthood [3], consequently results in a higher rate of mortality [1].

Dyslipidemia is highly prevalent among children [4]. One in every 250 children has familial hypercholesterolemia (FH), while about 1 in 5 children are overweight or obese, which is often associated with lipid abnormalities [5–7].

Dyslipidemia is defined as raised values of circulating total cholesterol (TC), low-density lipoprotein (LDL), non-high-density lipoprotein (non-HDL), triglycerides (TG), and reduced HDL [8]. Lipid profiling has an irreplaceable role in the assessment of dyslipidemia [9]. Previous studies suggested an independent relationship between childhood and adulthood serum lipid levels [10, 11].

The Expert Panel on Integrated Guidelines for Cardiovascular Health and Risk Reduction in Children and Adolescents provide the sufficient facts for universal lipid screening (TC, HDL, and non-HDL) for children and adolescents aged 9–11 years and again at 17–21 years for routine screening (Expert Panel on Integrated Guidelines for Cardiovascular Health and Risk Reduction in Children and Adolescents, [12]).

Identification of the reference value of blood lipid concentrations is critically obese for the accurate determination of children and adolescents with dyslipidemia. Reference values should be derived from healthy population [13]. Because lipid levels vary with age, sex, and ethnicity, age- and gender-specific, reference values for each region/ethnicity are needed [14].

Unfortunately, there are currently no up-to-date reference ranges that can be applied to accurately explain the results of laboratory tests for clinicians in the pediatric population [15].

Dyslipidemia prevalence as described in a national Iranian study is high (45.7%), remarkably with regard to low HDL-C and hypertriglyceridemia in children and adolescents [16]. This was confirmed by a systematic review in 2015 [17].

The diagnosis of children with dyslipidemia is notably essential for healthcare providers in establishing and releasing recommendations for the management and prevention of future CVD [18]. In the present study, we intended to provide reference intervals for lipid concentrations from a Kawar (a city south of Iran) cohort of healthy children and adolescents aged 9–18 years.

## 2. Methods

### 2.1. Study Population

This was a prospective cohort study of healthy Iranian children aged 9–18 years, which has been previously detailed [19]. 472 cases (234 girls and 238 boys) contributed to this study using a systematic random sample stratified by age. Our samples were chosen randomly from the community of elementary, guidance, and secondary schools according to student numbers in each age group. Children with an established medical condition (e.g., thyroid problems, diabetes, renal failure, and adrenal insufficiency) or a history of early or late puberty were excluded from the analysis. Moreover, children who were taking medications (such as anticonvulsants and steroids) were not included in the study. Our study was approved by the ethics committee of Shiraz University of Medical Sciences. All procedures were performed in the study involving human participants were in accordance with the Code of Ethics of the World Medical Association (Declaration of Helsinki as revised in 2013) for experiments involving humans. The written informed consent for the use of samples was obtained from all participants.

### 2.2. Dyslipidemia Criteria

Dyslipidemia was defined according to the National Heart, Lung, and Blood Institute (NHLBI) panel Definition 2011 about dyslipidemia guidelines in children, the cutoff points for lipids are as follows: for total cholesterol: acceptable: <170 mg/dL (4.4 mmol/L), borderline: 170–199 mg/dL (4.4–5.2 mmol/L), and abnormal: >200 mg/dL (5.2 mmol/L); for LDL: acceptable: <10 mg/dL (2.9 mmol/L), borderline: 110–129 mg/dL (2.9–3.4 mmol/L), and abnormal: >130 mg/dL (3.4 mmol/L); for non-HDL: acceptable: <120 mg/dL (3.1 mmol/L), borderline: 120–144 mg/dL (3.1–3.7 mmol/L), and abnormal: >145 mg/dL (3.8 mmol/L); for TG (children 0–9 years): acceptable: <75 mg/dL (0.8 mmol/L), borderline: 75–99 mg/dL (0.8–1.1 mmol/L), and abnormal: >100 mg/dL (1.1 mmol/L); for TG (adolescents 10–19 years): acceptable: <90 mg/dL (1 mmol/L), borderline: 90–129 mg/dL (1–1.5 mmol/L), and abnormal: >130 mg/dL (1.5 mmol/L); and for HDL: acceptable: >45 mg/dL (1.2 mmol/L), borderline: 40–45 mg/dL (1–1.2 mmol/L), and abnormal: <40 mg/dL (1 mmol/L).

### 2.3. Pubertal Stage

Puberty was assessed based on the Tanner stages during the visit for the dual-energyX-ray absorptiometry (DEXA) scan. Tanner stages 1 were considered prepubertal, 2 and 3 were considered early pubertal, and Tanner stages 4 and 5 were considered pubertal.

### 2.4. Lipid Measurements

All the samples from each participant were stored in the Shiraz Endocrinology Research Center after overnight fasting. The samples were collected at 8 a.m. Serum total cholesterol, HDL-C, and TG values were assessed by enzymatic reagents (Biosystems, Barcelona, Spain) with an A-25 Biosystem Autoanalyser. The Friedwald equation was used to indirectly measure LDL concentrations from calculated TG, HDLc, and TC [20]. Non-HDL-C was calculated by detracting HDL-C from total cholesterol [21]. The coefficient of variation (CV%) for TG, TC, and HDL methods was 1.7–2.6%, 1–1.9.5%, and 1.3–1.5%, respectively.

### 2.5. Statistical Analysis

Student's *t*-test, Mann–Whitney test, multivariate linear regression analysis, and Pearson correlation were run on SPSS V.18. The Lambda for skew, Mu for median, and Sigma for the generalized coefficient of variation (LMS) method were used to calculate smoothed BMI, cholesterol, TG, HDL, TC, LDL, and non-HDL curves for age centiles. LMS Chart Maker [22, 23], “Pan H, Cole TJ. User's Guide to LMS Chart Maker, Medical Research Council [24],” and Excel software were used to construct the gender-specific reference plots showing 3, 10, 25, 50, 75, 90, and 97th percentiles of BMI, cholesterol, TG, HDL, TC, LDL, and non-HDL.

## 3. Results

**Figure 1:**
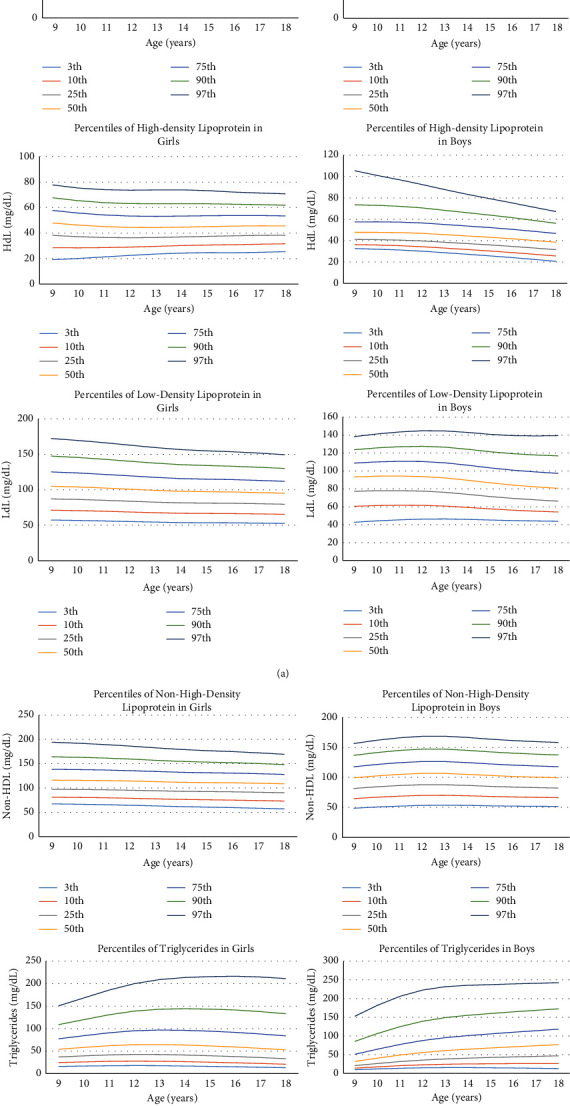
Percentile curves for fasting plasma concentrations of cholesterol, LDL, HDL, non-HDL, and TG in the population-based cohort. All of lipid concentrations are in mg/dL.

From a population-based cohort, 473 children and adolescents with an average age of 14 years (232 girls) were included in the study. According to the (NHLBI) panel Definition 2011, dyslipidemia prevalence in our population was as follows: for total cholesterol 7.2%, for LDL 8%, for non-HDL 10.5%, for TG 14.5%, and for HDL 32%. Based on the International Obesity Task Force (IOTF)/Coles index cutoffs, we found that 6.3% of boys and 5.5% of girls were overweight and only 0.4% of girls were obese. There were no significant differences between sexes and age, HDL and TG (*P* > 0.05). Descriptive information on the Kawar cohort is reported in Table 1. All comparisons were performed between boys and girls in the whole population.  Table 2 displays TC, LDL, HDL, non-HDL, and TG percentiles, and Figure 1 shows the percentile curves from the population-based cohort.

### 3.1. TC

The overall TC mean and SD values were 161.27 (28.44) mg/dl and 152.29 (32.41) mg/dl in girls and boys, respectively, with a significant gender difference (*P* = 0.001). Throughout the age range surveyed, boys' TCs have declined from about 12 years of age, reaching a minimum around 18 years of age. In girls, TC began to decline at age 11, and reached a minimum around age 18. The median of cholesterol in boys decreased from 151.2 to 137.9 and in girls from 161.8 to 155.0 in 9–18 years of age (*P* = 0.001). The percentile curves of cholesterol in boys and girls are in a similar manner and approximately constant.

### 3.2. TG

The overall TG mean value was 74.54 (48.15) mg/dl in girls and 76.62 (55.11) mg/dl in boys. The gender difference was not significant (*P* = 0.715). For the 3rd percentile in girls and boys, TG started dropping at the age of 12 with the lowest values reached at the age of 18. In the 97^th^ percentile for boys, TG showed an ascending trend from 9 to 18 years of age. For girls, TG increased from 9 to 14 years of age and decreased after that through 16 years of age. The median of TG in boys augmented from 32.0 to 71.1 and in girls from 54 to 59.2 in 9–18 years of age (*P* > 0.05). The TG percentile curves in boys and girls are in a similar manner and curves were up-sloping. The percentile 97^th^ curves diverged more from the 50th percentile curves. TG in boys was correlated with age (*r* = 0.23), BMI (*r* = 0.38), weight (*r* = 0.32), and waist circumference (*r* = 0.28) (*P* < 0.001), whereas in girls, it was negligible (*P* > 0.001).

### 3.3. HDL

The HDL overall mean value was 47.05 (15.37) mg/dl in girls and 47.40 (16.14) mg/dl in boys. The gender difference was not significant (*P* = 0.853). HDL values in girls in the 3rd percentile exhibited an ascending trend while it was descending in the 97^th^ percentile. For boys, the descending trend was observed in both percentiles. The median of HDL in boys declined from 47.8 to 41.8 and in girls from 47.9 to 45.5 in 9–18 years of age (*P* > 0.05). The HDL percentile curves in boys and girls are in a similar manner and nearly constant.

### 3.4. LDL

The LDL overall mean value was 99.35 (27.26) mg/dl in girls and 89.59 (25.25) mg/dl in boys. The gender difference was significant (*P* < 0.001). Similar to HDL, LDL in girls displayed a downward trend in the 3rd and 97^th^ percentiles. The median LDL in boys reduced from 93.3 to 84.2 and in girls from 105.1 to 96.9 in 9–18 years of age (*P* < 0.001). The LDL percentile curves in boys and girls are in a similar manner and almost constant.

### 3.5. Non-HDL

The non-HDL overall mean value of was 114.22 (29.73) mg/dl in girls and 104.88 (28.39) mg/dl in boys. The gender difference was significant (*P* < 0.001). Non-HDL in girls also displayed a downward trend in the 3rd and 97^th^ percentiles. Non-HDL in boys started dropping at the age of 13 with the lowest values around 18 years of age in both percentiles. The median non-HDL in boys elevated from 99.2 to 101.6 and in girls decreased from 116.2 to 110.7 at age 9–18 (*P* < 0.001). The trend of HDL percentile curves in boys and girls are similar.

### 3.6. Puberty

Data of the pubertal stages revealed that 41.47% (*N* = 88) of girls and 21.5% (*N* = 47) of boys were in the late pubertal stage. Late pubertal girls displayed lower TG, TC, and non-HDL values compared to mid-pubertal and early pubertal girls, while HDL values were higher. In boys, the late puberty stage was correlated with lower levels of LDL, TC, HDL, and non-HDL, but TG levels were higher. Linear regression analysis explored the simultaneous effects of age, sex, BMI, and puberty on lipid levels (Table 3). The results exhibited that puberty significantly affected the variation in TC, LDL, and non-HDL in all cases.

## 4. Discussion

This study presented the reference intervals for TG, HDL, TC, LDL, and non-HDL with age- and sex-specific percentiles for lipid and lipoprotein levels in a population-based cohort of Iranian children and adolescents. The reference intervals in this study were divided by sex, and *P* values were reported based on comparison between genders in the whole population. Age partitioning was only done for generating descriptive data in each age group and comparison was done between genders in the whole population. It can be used to indicate individuals with possible familial hypercholesterolemia (FH), which could be helpful for precluding CVD in later life. In addition, this might aid clinicians in using pharmacological therapy or changing lifestyle in children and adolescents.

The findings of our study demonstrated that concentrations of TC, LDL, and non-HDL were significantly higher in girls compared to boys. Our observations of higher TC and LDL-C levels in girls compared with boys were similar to the HELENA study in the European adolescents [25]; a study of healthy Indian school children and adolescents [26], and the CASPIAN-V study [18], while Lopez et al. did not observe any gender differences in the Fuenlabrada study [27]. In Brazilian school children and adolescents, girls also had greater values of TC and LDL-C compared to boys [28]. In Chinese children and adolescents, aged 7–18 years, we showed a higher prevalence of elevated TC, HDL, and LDL-C in girls than in boys [28]. These outcomes were similar to the principal national and international epidemiological studies demonstrating raised levels of all lipoproteins and lipids in female subjects, regardless of age or ethnicity [29, 30].

In the current study TG elevated with age in both genders, whereas HDL, TC, LDL, and non-HDL decreased. We observed that most of the changes in lipid values occurred before the age of 12, and after that, the trend of changes was constant. It seems that lipid levels fluctuate during the prepubertal stage, and at the mature/advanced puberty stage, there was no change in lipid concentrations.

According to NCEP Expert Panel on Cholesterol Levels in Children, the defined criteria for dyslipidemia are as follows: TC ≥ 200 mg/dL, LDL-C ≥ 130 mg/dL, HDL-C < 40 mg/dL, and non-HDLC ≥ 145 mg/dL. Furthermore, the recommended thresholds for defining hypertriglyceridemia have been ≥100 mg/dL and ≥130 mg/dL in children aged 0–9 and 10–19 years, respectively [31]. In the present study, the 97^th^ percentile for TG, HDL, TC, LDL, and non-HDL was higher than the values recommended by the NCEP.

Previously, two studies have reported the lipid reference values in Iranian children and adolescents [18, 32]. In comparison with the CASPIAN-V study, the mean values of HDL in both genders, TC, non-HDL, and LDL in girls were higher and TG was lower in the present study. In comparison with the study that was conducted in Birjand, a city in the east of Iran, LDL in both genders and TC in girls was higher compared to LDL and TC values in the current study, but HDL and TG were lower in the Birjand study compared to our study. These differences between different regions of Iran may be due to dietary regimen, physical activity, hormonal status, environmental factors, and socioeconomic status, which can influence the lipid profile in children [33, 34] so that it is vital to provide age- and gender-specific reference intervals using a local population [1].

In the current study, we observed that puberty was associated with better lipid values in both genders except for TG levels in boys. The effects of puberty on lipid concentrations were similar to the effects of age in our population. During the pubertal period, due to increased levels of estrogen and progesterone in girls and by increased testosterone levels among boys, the cholesterol is shifted into the growing cells, which results in decreased lipid levels [35]. Similar to our results, Bertrais et al. [36] described that prepubertal children have greater mean levels of TC and TG than those at mature/advanced puberty stage. Also, Eissa et al. [37] stated decreasing levels of TC and non-HDL-C during puberty, with differences according to sex and race. In this study, regression analysis exhibited that puberty was significantly associated with changes in TC, LDL, and non-HDL through the studied population.

The strength of our study was that, in contrast to two previous studies executed in this region with the lack of data on puberty, we explored the effect of puberty on lipid levels in Iranian children and adolescents aged 9–18 years. Considering pubertal stage in providing lipid reference values for children and adolescents may counter the underdiagnose dyslipidemia in some cases. Also, in this study, the measurement of lipid levels was executed on new samples in a single central laboratory in order to avoid the variation in the analytical methods. The limitation of our study is that due to dietary and geographical diversity in Iran, our study population is not illustrative of the Iranian pediatric population in general. Another limitation was a relatively small sample size.

## 5. Conclusion

Our study provides age- and sex-specific reference values for plasma lipid and lipoprotein levels in Iranian children and adolescents. We expect that these lipid and lipoprotein values, translated into age- and sex-specific percentiles, can assist clinicians as an effective and consistent tool to help identify children and adolescents with dyslipidemia.

## Figures and Tables

**Table 1:** Descriptive information on the population-based cohort. The *P* values were tested by student's *t*-test, Mann–Whitney test, and Chi-square. All of lipid concentrations are in mg/dL P values > 0.05 are shown in bold.

Age	Number (%) F	Number (%) M	BMI (F)	BMI (M)	Waist circumference (cm) F	Waist circumference (cm) M	TG (F)	TG (M)	HDL (F)	HDL (M)	Chol (F)	Chol (M)	LDL (F)	LDL (M)	Non-HDL(F)	Non-HDL(M)
9	9 (3.9)	11 (4.6)	15.7 ± 2.2	15.0 ± 1.5	57.7 ± 6.1	55.5 ± 3.9	50.9 ± 24.1	28.9 ± 16.6	67.1 ± 48.8	44.8 ± 8.7	169.4 ± 28.8	129.8 ± 11.2	92.2 ± 54.6	79.6 ± 12.7	102.4 ± 58.0	84.9 ± 11.5
10	27 (11.6)	22 (9.1)	15.6 ± 2.0	16.1 ± 1.9	58.9 ± 5.4	60.3 ± 7.0	60.3 ± 36.1	54.6 ± 49.9	44.2 ± 13.4	50.1 ± 12.4	164.6 ± 29.8	162.1 ± 38.2	108.3 ± 24.5	101.1 ± 29.4	120.4 ± 26.0	112.0 ± 31.6
11	26 (11.2)	24 (9.9)	15.2 ± 1.6	15.9 ± 1.9	60.8 ± 5.4	60.2 ± 7.0	86.8 ± 60.1	59.7 ± 30.1	45.5 ± 12.3	53.0 ± 21.0	164.8 ± 31.2	153.7 ± 24.3	101.9 ± 31.2	88.8 ± 14.8	119.3 ± 34.0	100.7 ± 17.1
12	26 (11.2)	23 (9.5)	16.0 ± 2.0	16.4 ± 2.4	65.1 ± 7.9	61.7 ± 7.9	87.5 ± 44.3	83.2 ± 68.5	46.7 ± 9.0	57.0 ± 13.7	163.2 ± 27.7	172.3 ± 30.6	99.0 ± 28.9	98.7 ± 23.0	116.5 ± 29.8	115.2 ± 29.6
13	26 (11.2)	25 (10.3)	17.3 ± 2.2	16.9 ± 2.7	69.5 ± 6.4	64.0 ± 8.5	76.0 ± 41.9	83.0 ± 57.6	45.4 ± 10.7	45.5 ± 14.2	159.3 ± 30.4	160.8 ± 40.3	98.7 ± 26.0	98.7 ± 30.9	113.9 ± 28.4	115.3 ± 34.1
14	27 (11.6)	34 (14.1)	19.0 ± 2.7	17.1 ± 3.0	72.2 ± 6.5	65.9 ± 9.0	85.5 ± 51.9	81.1 ± 56.2	45.8 ± 9.7	47.9 ± 24.9	154.6 ± 24.6	153.7 ± 29.0	91.7 ± 20.1	89.6 ± 27.8	108.8 ± 22.9	105.8 ± 30.6
15	22 (9.4)	32 (13.3)	19.0 ± 2.4	18.4 ± 2.8	75.6 ± 8.7	72.2 ± 9.0	64.3 ± 51.0	73.7 ± 53.9	49.1 ± 17.1	47.4 ± 12.9	159.1 ± 27.2	147.0 ± 31.2	97.2 ± 23.9	84.8 ± 23.4	110.0 ± 23.1	99.6 ± 28.5
16	23 (9.9)	28 (11.6)	20.8 ± 3.4	19.0 ± 2.9	79.6 ± 8.8	75.9 ± 7.8	82.3 ± 51.0	98.3 ± 68.2	44.7 ± 13.9	45.1 ± 10.4	170.6 ± 31.8	146.5 ± 31.0	109.4 ± 25.4	81.7 ± 25.2	125.8 ± 31.4	101.4 ± 28.5
17	22 (9.4)	20 (8.3)	19.2 ± 4.3	20.1 ± 4.1	78.2 ± 9.2	79.7 ± 8.9	62.3 ± 44.9	80.6 ± 39.1	50.3 ± 10.1	41.6 ± 11.8	155.8 ± 26.1	142.4 ± 32.7	93.1 ± 25.4	84.8 ± 24.7	105.5 ± 29.9	100.9 ± 27.3
18	24 (10.3)	22 (9.1)	20.0 ± 2.6	19.4 ± 3.0	74.0 ± 8.0	75.4 ± 8.9	70.9 ± 51.1	90.9 ± 52.3	45.2 ± 10.4	39.4 ± 11.6	156.3 ± 26.9	142.6 ± 29.2	96.9 ± 22.9	85.0 ± 22.6	111.0 ± 24.3	103.2 ± 24.1
*P* value	**0.679**		**0.306**		**0.059**		**0.715**		**0.853**		**0.001**		**<0.001**		**<0.001**	

F: female and M: male

**Table 2:** Percentiles for fasting plasma concentrations of cholesterol, LDL, HDL, non-HDL, and TG in the population-based cohort. The 3rd and 97th percentiles for each age group in girls and boys are presented. There was significant difference between the 3rd and 97th percentiles (highlighted in grey). All of lipid concentrations are in mg/dL.

Age	*BMI*				*TG*				*HDL*				*Chol*				*LDL*				*Non-HDL*			
	3th percentile		97th percentile		3th percentile		97th percentile		3th percentile		97th percentile		3th percentile		97th percentile		3th percentile		97th percentile		3th percentile		97th percentile	
	Girls	Boys	Girls	Boys	Girls	Boys	Girls	Boys	Girls	Boys	Girls	Boys	Girls	Boys	Girls	Boys	Girls	Boys	Girls	Boys	Girls	Boys	Girls	Boys
9	12.22	12.97	19.00	19.18	16.05	9.81	150.61	152.71	19.14	32.58	77.73	105.42	126.44	95.54	242.09	213.29	57.36	42.62	172.28	138.25	67.39	48.54	194.29	156.45
10	12.41	13.13	19.78	20.05	16.89	11.99	168.11	182.05	20.12	32.03	75.32	101.05	124.52	98.42	237.62	218.46	56.83	44.20	169.65	141.25	66.65	50.54	192.11	161.84
11	12.64	13.27	20.65	20.90	17.58	13.78	185.61	206.07	21.34	31.27	74.00	96.98	122.46	100.61	233.41	222.21	56.12	45.40	166.50	143.48	65.72	52.15	189.54	166.01
12	12.98	13.40	21.72	21.78	17.85	14.94	199.93	222.68	22.54	30.17	73.62	92.59	120.13	101.72	229.18	223.67	55.26	46.18	162.98	144.80	64.51	53.27	186.30	168.58
13	13.40	13.57	22.98	22.74	17.59	15.45	208.95	231.73	23.56	28.76	73.80	87.86	117.67	101.42	225.11	222.17	54.42	46.34	159.52	144.63	63.10	53.62	182.64	168.70
14	13.81	13.82	24.25	23.85	16.95	15.44	213.59	235.58	24.30	27.29	73.86	83.46	115.31	100.06	221.73	218.49	53.77	45.89	156.68	142.94	61.75	53.24	179.27	166.59
15	14.12	14.13	25.43	25.09	16.12	15.11	215.67	237.31	24.63	25.82	73.28	79.45	113.32	98.30	219.49	214.08	53.49	45.17	154.94	140.81	60.69	52.61	176.90	163.73
16	14.31	14.45	26.44	26.38	15.24	14.61	216.45	239.18	24.59	24.23	72.13	75.44	111.28	96.63	217.64	210.00	53.35	44.56	153.60	139.41	59.72	52.12	174.94	161.37
17	14.36	14.74	27.29	27.65	14.23	13.90	214.70	240.71	24.91	22.48	71.42	71.30	108.69	95.12	215.27	206.39	53.00	44.18	151.68	139.07	58.46	51.83	172.27	159.63
18	14.32	15.01	28.07	28.89	13.17	13.00	211.32	242.48	25.43	20.67	70.75	67.23	105.64	93.71	212.58	203.11	52.55	43.86	149.50	139.34	57.10	51.62	169.43	158.21

**Table 3:** Linear regression analysis for the effect of age, sex, stage of puberty, and BMI on lipid profile. *P* values less than 0.05 are shown in bold. All of lipid concentrations are in mg/dL.

Data	*TG*			*HDL*			*Chol*			*Non-HDL*			*LDL*		
	*β*	SE	*P*value	*β*	SE	*P*value	*β*	SE	*P*value	*β*	SE	*P*value	*β*	SE	*P*value
Age	1.126	2.061	0.585	−1.082	0.683	0.114	1.058	1.264	0.403	2.140	1.208	0.077	1.892	1.103	0.087
Sex	1.866	4.948	0.993	0.817	1.640	0.618	−11.958	3.035	<0.001	−12.775	2.901	<0.001	−13.135	2.648	<0.001
Puberty	−3.875	3.563	0.277	1.304	1.181	0.270	−6.781	2.186	0.002	−8.084	2.089	<0.001	−7.275	1.907	<0.001
BMI	5.092	0.879	<0.001	−0.440	0.291	0.132	1.998	0.540	<0.001	2.438	0.516	<0.001	1.415	0.471	0.003

## Data Availability

The datasets used and/or analyzed during the current study are available from the corresponding author on reasonable request.
